# Functional and Activation Profiles of Mucosal-Associated Invariant T Cells in Patients With Tuberculosis and HIV in a High Endemic Setting

**DOI:** 10.3389/fimmu.2021.648216

**Published:** 2021-03-22

**Authors:** Avuyonke Balfour, Charlotte Schutz, Rene Goliath, Katalin A. Wilkinson, Sumaya Sayed, Bianca Sossen, Jean-Paul Kanyik, Amy Ward, Rhandzu Ndzhukule, Anele Gela, David M. Lewinsohn, Deborah A. Lewinsohn, Graeme Meintjes, Muki Shey

**Affiliations:** ^1^Department of Medicine, Faculty of Health Sciences, University of Cape Town, Cape Town, South Africa; ^2^Wellcome Centre for Infectious Diseases Research in Africa (CIDRI-Africa), Faculty of Health Sciences, Institute of Infectious Disease and Molecular Medicine (IDM), University of Cape Town, Cape Town, South Africa; ^3^The Francis Crick Institute, London, United Kingdom; ^4^South African Tuberculosis Vaccine Initiative, Faculty of Health Sciences, Institute of Infectious Disease and Molecular Medicine (IDM), University of Cape Town, Cape Town, South Africa; ^5^Division of Pulmonary and Critical Care Medicine, Department of Medicine, Oregon Health and Science University, Portland, OR, United States; ^6^Division of Infectious Diseases, Department of Paediatrics, Oregon Health and Science University, Portland, OR, United States

**Keywords:** MAIT cell, MAIT activation, human immunodeficiency virus, tuberculosis, MAIT cell function

## Abstract

**Background:** MAIT cells are non-classically restricted T lymphocytes that recognize and rapidly respond to microbial metabolites or cytokines and have the capacity to kill bacteria-infected cells. Circulating MAIT cell numbers generally decrease in patients with active TB and HIV infection, but findings regarding functional changes differ.

**Methods:** We conducted a cross-sectional study on the effect of HIV, TB, and HIV-associated TB (HIV-TB) on MAIT cell frequencies, activation and functional profile in a high TB endemic setting in South Africa. Blood was collected from (i) healthy controls (HC, *n* = 26), 24 of whom had LTBI, (ii) individuals with active TB (aTB, *n* = 36), (iii) individuals with HIV infection (HIV, *n* = 50), 37 of whom had LTBI, and (iv) individuals with HIV-associated TB (HIV-TB, *n* = 26). All TB participants were newly diagnosed and sampled before treatment, additional samples were also collected from 18 participants in the aTB group after 10 weeks of TB treatment. Peripheral blood mononuclear cells (PBMC) stimulated with BCG-expressing GFP (BCG-GFP) and heat-killed (HK) *Mycobacterium tuberculosis* (*M.tb*) were analyzed using flow cytometry. MAIT cells were defined as CD3^+^ CD161^+^ Vα7.2^+^ T cells.

**Results:** Circulating MAIT cell frequencies were depleted in individuals with HIV infection (*p* = 0.009). MAIT cells showed reduced CD107a expression in aTB (*p* = 0.006), and reduced IFNγ expression in aTB (*p* < 0.001) and in HIV-TB (*p* < 0.001) in response to BCG-GFP stimulation. This functional impairment was coupled with a significant increase in activation (defined by HLA-DR expression) in resting MAIT cells from HIV (*p* < 0.001), aTB (*p* = 0.019), and HIV-TB (*p* = 0.005) patients, and higher HLA-DR expression in MAIT cells expressing IFNγ in aTB (*p* = 0.009) and HIV-TB (*p* = 0.002) after stimulation with BCG-GFP and HK-*M.tb*. After 10 weeks of TB treatment, there was reversion in the observed functional impairment in total MAIT cells, with increases in CD107a (*p* = 0.020) and IFNγ (*p* = 0.010) expression.

**Conclusions:** Frequencies and functional profile of MAIT cells in response to mycobacterial stimulation are significantly decreased in HIV infected persons, active TB and HIV-associated TB, with a concomitant increase in MAIT cell activation. These alterations may reduce the capacity of MAIT cells to play a protective role in the immune response to these two pathogens.

## Introduction

Tuberculosis (TB) is one of the deadliest infectious diseases globally, with an estimated 10 million new cases and 1.4 million deaths in the year 2019 (1). South Africa has a high TB burden and accounts for about 3.6% of the global cases, with 360,000 cases of TB, and 58,000 deaths due to TB annually (1). One of the main drivers behind this high TB incidence is the Human Immunodeficiency Virus (HIV). South Africa had an estimated 7.5 million people living with HIV and reported about 200,000 new HIV infections in 2019 (2, 3). Of the total TB cases in 2019 in South Africa, 58% were from HIV infected people, these individuals had a disproportionally larger mortality rate of 62 (per 100,000 population) compared to HIV-negative individuals who had a mortality rate of 38 (1).

HIV and TB are both associated with impairment of the immune system (4, 5). HIV infection results in the depletion and functional impairment of CD4 T cells that are crucial to the containment of *Mycobacterium tuberculosis* (6, 7). TB results in lymphopaenia and more rapid progression of untreated HIV infection and HIV-induced depletion of CD4 T cells due to immune activation (5, 7). T cell immunity is therefore central in the pathogenesis of both infections.

Mucosal-associated invariant T (MAIT) cells have been shown to play a critical role in antibacterial immunity (8, 9). MAIT cells are an evolutionarily conserved, non-conventional, and innate-like subset of T cells that express a semi-invariant T cell receptor, TRAV1-2 (Vα7.2). This semi-invariant T cell receptor enables MAIT cells to recognize MHC-related protein 1 (MR1)-bound microbial vitamin metabolites, principally those derived from riboflavin pathways that are present in microbes but not in humans (10). MAIT cells can be identified through the expression of a variety of other markers such as IL-12R, IL-18R (11), the C-type lectin receptor, CD161, and CD26 (12). More recently, MR1 tetramers have been used to identify MAIT cells (13, 14). MAIT cells can be sub-divided into 3 different subsets; the predominant CD8+ subset, the CD4-, CD8-(double negative, DN) subset, and the CD4+ subset which has the lowest frequency (15).

Numerous studies have demonstrated that MAIT cells are responsive to bacteria-infected antigen presenting cells (APCs) (8, 9, 16). Chua et al. (8) demonstrated that MAIT cells have the capacity to inhibit intracellular bacterial growth in BCG-infected macrophages. Following antigen recognition, MAIT cells are activated and can rapidly produce a variety of cytokines such as IFNγ and TNF-α (16, 17). In addition, MAIT cells also have the capacity to produce cytotoxic and cytolytic molecules such as granzyme B and perforin (18), thereby having capacity to kill infected cells (9). *In vivo*, the absence of MR1 (and therefore MAIT cells) results in failure to control infection as mice lacking MR1 have been shown to have higher bacterial burden in their spleen and lungs compared to wild-type mice (8, 19, 20).

Studies that investigated the effect of HIV and TB disease on MAIT cells have shown decreased cell frequencies in both TB disease (21, 22) and chronic HIV infection (23–25). In TB disease, MAIT cells have also been shown to be functionally impaired with significantly lower expression of IFNγ, TNF-α, IL-17, and granzyme B (18). In HIV however, findings on MAIT cell functions have been inconsistent. Leeasnyah et al. (23) showed reduced frequencies of MAIT cells expressing IFNγ, TNF-α, and IL-17 in people with HIV-1 infection, while Fernandez observed no significant differences in frequencies of MAIT cells expressing IFNγ and TNF-α (25).

The impact of anti-retroviral treatment (ART) on MAIT cells has been investigated in several studies. Leeansyah et al. (23) and Wong et al. (22), found that the frequencies of MAIT cells were not fully restored by long-term ART and that there was only partial restoration of the capacity of MAIT cells to express IFNγ, TNF-α, and IL-17 (23). Concentrations of these cytokines were higher in individuals on ART, but still lower than healthy uninfected controls (23). There are no published studies reporting the effect of TB treatment on MAIT cell frequencies and functions.

We evaluated the effect of HIV, TB, and HIV-associated TB on the frequencies and function of MAIT cells, including in different MAIT cell subsets. We also investigated the effect of the first 10 weeks of TB treatment on MAIT cell frequencies and function.

## Methods and Materials

### Study Participants

Blood was collected from 138 participants recruited at Ubuntu HIV-TB Clinic, Khayelitsha, Cape Town, South Africa. Of these, 26 were HIV negative TB negative healthy controls (HC), 92% of whom had latent TB infection (LTBI), 50 were HIV positive TB negative and 70% had LTBI (HIV), 36 were HIV negative TB positive (aTB), and 26 had HIV-associated TB (HIV-TB). All study participants were screened for TB symptoms and had chest radiographs conducted. HC and HIV group participants were asymptomatic and had normal Chest X-rays. Active TB was confirmed or excluded by sputum culture and GeneXpert. We assessed healthy controls and HIV group participants for TB exposure using QuantiFERON-TB Gold Plus assay. Participants were considered to have latent infection if the IFNγ concentrations were ≥0.35 IU/ml, and without latent infection if IFNγ concentrations were <0.35 IU/ml. Participants included into the 2 TB groups (aTB and HIV-TB) were recruited into the study before the start of TB treatment (or maximum of 3 doses). The study exclusion criteria included: declining HIV testing, pregnancy, having received more than three doses of TB treatment, presence of symptomatic anemia, and asthma or chronic obstructive pulmonary diseases. A subset of 18 individuals in the active TB group was chosen for repeat blood sample collection after 6–10 weeks (first 10 weeks) of TB treatment. Written informed consent was obtained from all study participants. Ethical approval for this study was obtained from the Faculty of Health Sciences Human Research Ethics Committee of the University of Cape Town (HREC Ref: 011/2017).

### Sample Processing

Blood samples were collected into Heparinized blood collection tubes. Peripheral blood mononuclear cells (PBMC) were isolated from blood *via* density gradient centrifugation with the Ficoll-Paque method and cryopreserved in fetal calf serum (FCS) containing 10% dimethyl sulfoxide (DMSO). These were then frozen at −80°C overnight and transferred to liquid nitrogen for long-term storage.

### Cell Stimulations

Cryopreserved PBMC were thawed and rested in R10 media (10% FBS, 1% Penicillin/Streptomycin, 1% HEPES buffer, and 50 mM 2-β-mercaptoethanol) at 37°C and 5% CO_2_ for 6 h prior to conducting stimulation assays. Following resting, cells were counted, and 1 million PBMC were stimulated with heat killed-*M.tb* (HK-*M.tb)* (5 × 10^5^ CFU/ml), live *Mycobacterium bovis* BCG (1 × 10^6^ and 5 × 10^6^ CFU/million cells, using BCG multiplicity of infection (MOI) = 1 (BCG 1) and BCG MOI= 5 (BCG 5), respectively) and PHA (4 μg/ml). For the background/unstimulated control, R10 media was used. Samples were incubated at 37°C and 5% CO_2_ for 18 h, following which, 100 μl supernatant was collected, transferred into Sarstedt tubes and stored at −80°C. Cells were then restimulated with respective antigen cocktails with the addition of anti-CD107a antibody and Monensin + Brefeldin and incubated for an additional 6 h at 37°C at 5% CO_2_.

### Flow Cytometry

The following antibodies were used: CD161-BV605 (HP-3G10, BioLegend), CD11c-BV650 (clone B-ly6, BD Biosciences), CD107a-BV711 (clone H4A3, BioLegend), CD4-BV786 (clone SK3, BD Biosciences), Vα7.2-AF-647 (clone 3C10, BioLegend), HLA-DR-AF-700 (clone L243, BioLegend), CD8-APC-H7 (clone SK1, BD Biosciences), IFNγ-V450 (clone B27, BD Biosciences), CD40-PerCp-Cy5.5 (clone 53C, BioLegend), CD3-ECD (clone UCHT1, Beckman Coulter), and PD1-BV711 (Clone EH12.2H7, BioLegend). Aqua fluorescent dye (Invitrogen) was used as a viability dye.

Following stimulation, cells were stained for viability with the aqua fluorescent dye and incubated for 10 min at room temperature. After viability staining, surface staining was performed with CD161, Vα7.2, CD4, and CD40. Cells were then fixed and permeabilized with Cytofix/Cytoperm buffer (BD Biosciences) followed by intracellular staining with CD3, CD8, CD14, CD11c, HLA-DR, and IFNγ. The stained samples were acquired on a BD LSR Fortessa FACSDiva Software. A total of 500,000 events were collected and results analyzed using FlowJo (version 9.9.6, FlowJo LLC). We defined MAIT cells as CD3^+^CD161^+^Vα7.2^+^ T cells, after excluding doublets and dead cells.

### MR1 Tetramer Staining

For more precise identification of MAIT cells, and to validate the findings that we observed using antibodies, we used MR1 tetramers to investigate MAIT cell frequencies. For this, an MR1 tetramer conjugated to Allophycocyanin (APC), and loaded with 5-(2-oxopropylideneamino)-6-D-ribitylaminouracil (5-OP-RU), which is a known potent MAIT cell activating ligand, was used (Obtained from NIH Tetramer Core Facility, Emory University, Atlanta, GA, United States). PBMC were thawed and rested for 2 h, counted, and stained for viability. Cells were then stained with 50 μl of 5-OP-RU-loaded MR1 tetramer (diluted 1:100) at 37°C for 30 min. As a control, cells were stained with 6-formyl pterin (PF) loaded MR1 tetramer (diluted 1:100). Cells were washed and stained extracellularly with CD161, CD3, CD4, CD8, PD1, and CD14. Following staining, cells were washed and fixed in 1% paraformaldehyde and acquired using a BD LSR flow cytometer.

### Statistical Analysis

Data visualization and statistical analyses were performed using GraphPad Prism (V.8.4.2, GraphPad Software). For statistical comparisons between the groups, the non-parametric Kruskal-Wallis test was used with a Dunn's test to correct for multiple comparisons. Wilcoxon signed rank tests were used for paired samples. IBM SPSS (version 27.0.1.0) was used for analysis of covariance (ANCOVA) to adjust for sex differences and Bonferroni tests for multiple comparisons. A *p*-value <0.05 was considered statistically significant. Spearman correlation analyses were used to analyze relationships between variables.

We used t-Stochastic Neighbor Embedding (t-SNE) in order to visualize the cell subset distribution and functional distribution of MAIT cells. Flow cytometry files for individual participants belonging to the same group were concatenated into one file for each group, the individual group files were then downsampled to obtain an equal number of events across the four groups, these files were then further concatenated into a single file for t-SNE. The following markers were used for T cell frequencies and MAIT cell functions; CD4, CD8, CD107a, HLA-DR, and IFNγ. For MAIT cell subset frequencies, CD4, CD8, HLA-DR, CD107a, and IFNγ were used.

## Results

### Participant Characteristics

A total of 138 study participants were enrolled into the study, with patient characteristics summarized in Table 1. There were higher proportions of female participants in our HC (73.1%) and HIV group (76%) and a disproportionally large proportion of males in the aTB group (80.6%). TB negative healthy controls were tested for TB exposure, 92% had positive QFT results suggesting latent TB infection (LTBI) and of the 48 HIV positive TB negative group participants that were tested for QFT, 70% were positive (Table 1).

**Table 1:** Participant demographics and clinical characteristics.

	**Healthy controls (HC)**	**HIV only (HIV)**	**Active TB (aTB)**	**HIV-associated TB**	***p*-value**
*n*	26	50	36	26	
Females	19 (73.1 %)	38 (76.0%)	7 (19.4%)	14 (53.4%)	Chi-squared <0.001
Age, years	34 (26–40)	37 (31–43)	35 (24–43)	39 (31–44)	Kruskal-Wallis <0.112
CD4, cells/μl	ND	484 (333–541)	ND	222 (107–323)	Mann-Whitney <0.001
HIV VL, log10 copies/ml	ND	4.3 (3–5)	ND	4.7 (3.4–5)	Mann-Whitney 0.352
On ART:	NA	33 (66%)	NA	17 (65%)	
Yes		14 (28%)		7 (27%)	
No		3 (6%)		2 (8%)	
Unknown					
^*^QFT+	24 (92%)	34 (70%)	ND	ND	
QFT–	2 (8%)	14 (30%)	ND	ND	

* *No QFT results for 2 participants*.

*Categorical variables presented as count and percentage, n (%)*.

*Continuous variables presented as median with IQR: median [IQR]*.

*NA, Not applicable; ND, Not Done; QFT, QuantiFERON; IQR, Interquartile range*.

### Frequencies of MAIT Cells Are Reduced in HIV Infection and During Active TB

**Figure 1:**
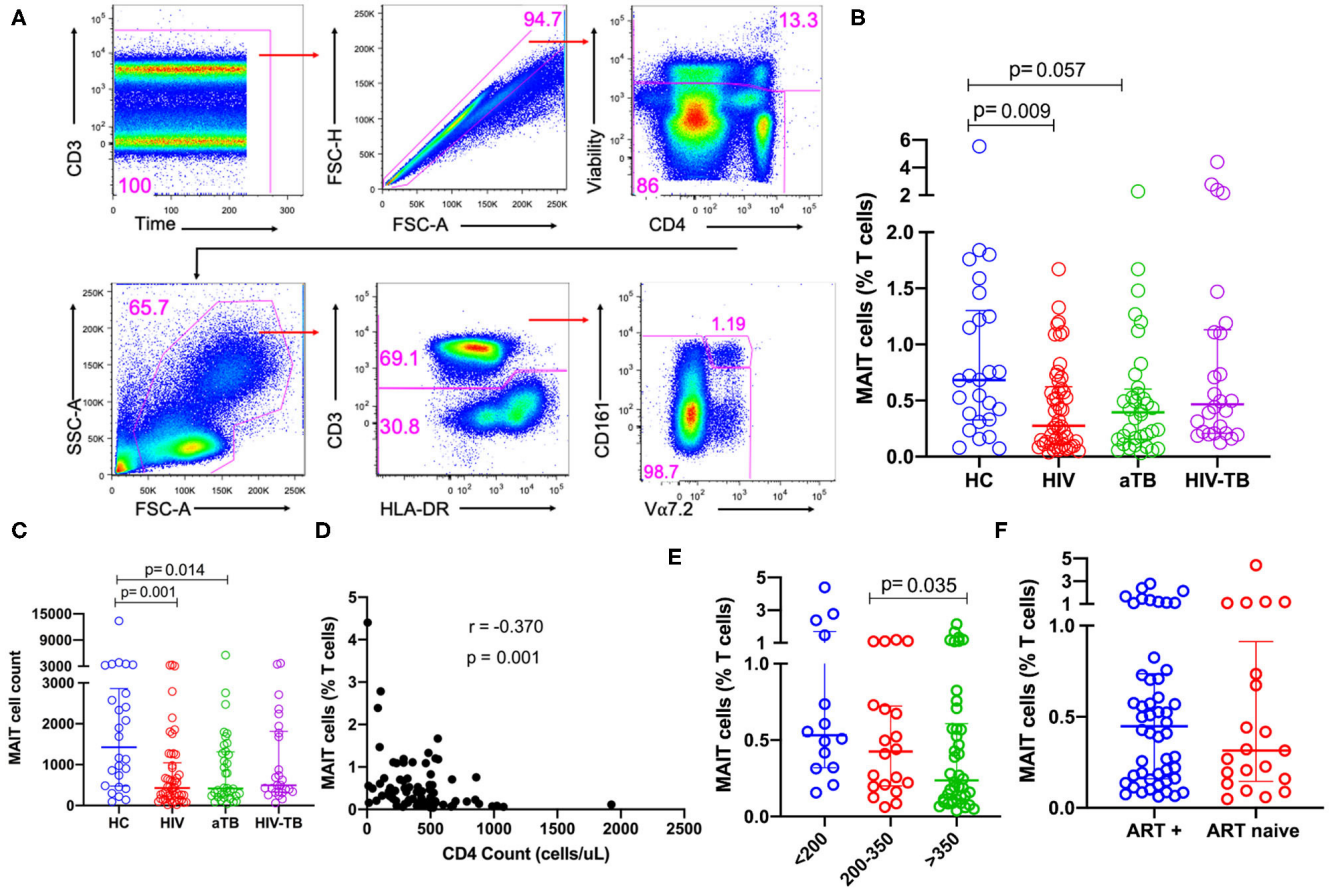
Frequencies of MAIT cells in patients with HIV infection, active TB, and HIV-associated TB. **(A)** Gating strategy used to identify MAIT cells. First, doublets and dead cells were excluded, then leukocytes selected, followed by CD3^+^ T cells. From the CD3^+^ T cells, MAIT cells were identified. **(B)** Frequencies of MAIT cells (%CD3 T cells) in HC, HIV, aTB, and HIV-TB. **(C)** MAIT cell counts (number of events in MAIT cell gate) in HC, HIV, aTB, and HIV-TB. **(D)** Relationship between MAIT cell frequencies and CD4 counts of HIV and HIV-TB group participants was investigated using Spearman rank correlation. **(E)** Effect of CD4 counts; and **(F)** ART on MAIT cell frequencies. *P*-values reported from Kruskal-Wallis test with a Dunn's *post-hoc* test for multiple comparisons and *p* < 0.05 reported as statistically significant. HC, Healthy controls group; HIV, HIV only group; aTB, active TB only group; HIV-TB, HIV-associated TB group.

We first sought to understand how HIV, TB and HIV-associated TB affect the frequencies of MAIT cells in blood. MAIT cells, defined as CD3^+^CD161^+^Vα7.2^+^ T cells, were identified using the gating strategy summarized in Figure 1A.

There were lower frequencies of MAIT cells in the HIV group (*p* = 0.009) compared to HC and a trend toward lower frequencies in the aTB group (*p* = 0.057) (Figure 1B). No significant differences were observed between the frequencies of MAIT cells in people with HIV-associated TB and healthy controls (Figure 1B). Looking at the numbers of MAIT cells (numbers of events in MAIT cell gate) showed a similar trend to MAIT cell frequencies, we observed lower numbers of MAIT cells in HIV (*p* = 0.001) and aTB (*p* = 0.014) compared to HC (Figure 1C). There was no significant difference in the number of MAIT cells between HIV-TB group and HC (Figure 1C). Supplementary Figure 1A shows that CD4 T cells are reduced from 39.7% in HC to 28.9% in the HIV group, and to 21.0% in the HIV-TB group. At the same time, CD8 T cells expanded from 33.2% in HC to 50.7% in the HIV group and 57.7% in the HIV-TB group.

We wanted to confirm whether our phenotypic analysis correlated with the now available MR1 tetramer, so we assessed MAIT cell frequencies using MR1-5-OP-RU in a subset of 10 HC, 10 HIV, 10 aTB, and 5 HIV-TB participants. Supplementary Figure 1B shows representative flow plots of MAIT cells identified with MR1-5-OP-RU tetramers. We found significantly lower MAIT cells in the aTB group compared to HC (*p* = 0.025) (Supplementary Figure 1C) while there were no significant differences between other groups. We found a strong positive correlation between the Vα7.2 antibody-identified MAIT cells and the MR1-5-OP-RU identified MAIT cells (*r* = 0.7009, *p* < 0.0001), (Supplementary Figure 1D). Supplementary Figure 1E shows the representative flow plots of MAIT cells identified with Vα7.2 antibody (top) and MR1-5-OP-RU tetramer (bottom). From these, we did not observe any significant differences between MAIT cells identified with Vα7.2 antibody and MAIT cells identified with MR1-5-OP-RU tetramer (Supplementary Figure 1F).

In the pooled HIV and HIV-TB groups, we assessed the effect of CD4 count, HIV VL and current ART status on MAIT cells. A significant but weak negative correlation was found between MAIT cell frequencies and CD4 counts (*p* = 0.001, *r* = −0.370) (Figure 1D), but frequencies were not correlated with HIV VL. We found that participants with CD4 counts above 350 cells/μl had significantly lower MAIT cell frequencies than participants with CD4 counts below 200 cells/μl (*p* = 0.035) (Figure 1E). We did not see any significant difference in MAIT cell frequencies between people on ART and those not on ART (Figure 1F), and found no difference in MAIT cell frequencies in people with HIV VL loads below 1,000 copies/ml and those with HIV VL above 1,000 copies/ml (data not shown). We evaluated dead cells to understand whether MAIT cells were preferentially dying and investigated CD161 downregulation by gating on the CD161^−^Vα7.2^+^ T cell population. We did not observe any significant differences in CD161 downregulation and cell death (data not shown).

### Evaluation of MAIT Cell Subsets in HIV Infection, Active TB, and HIV-Associated TB

**Figure 2:**
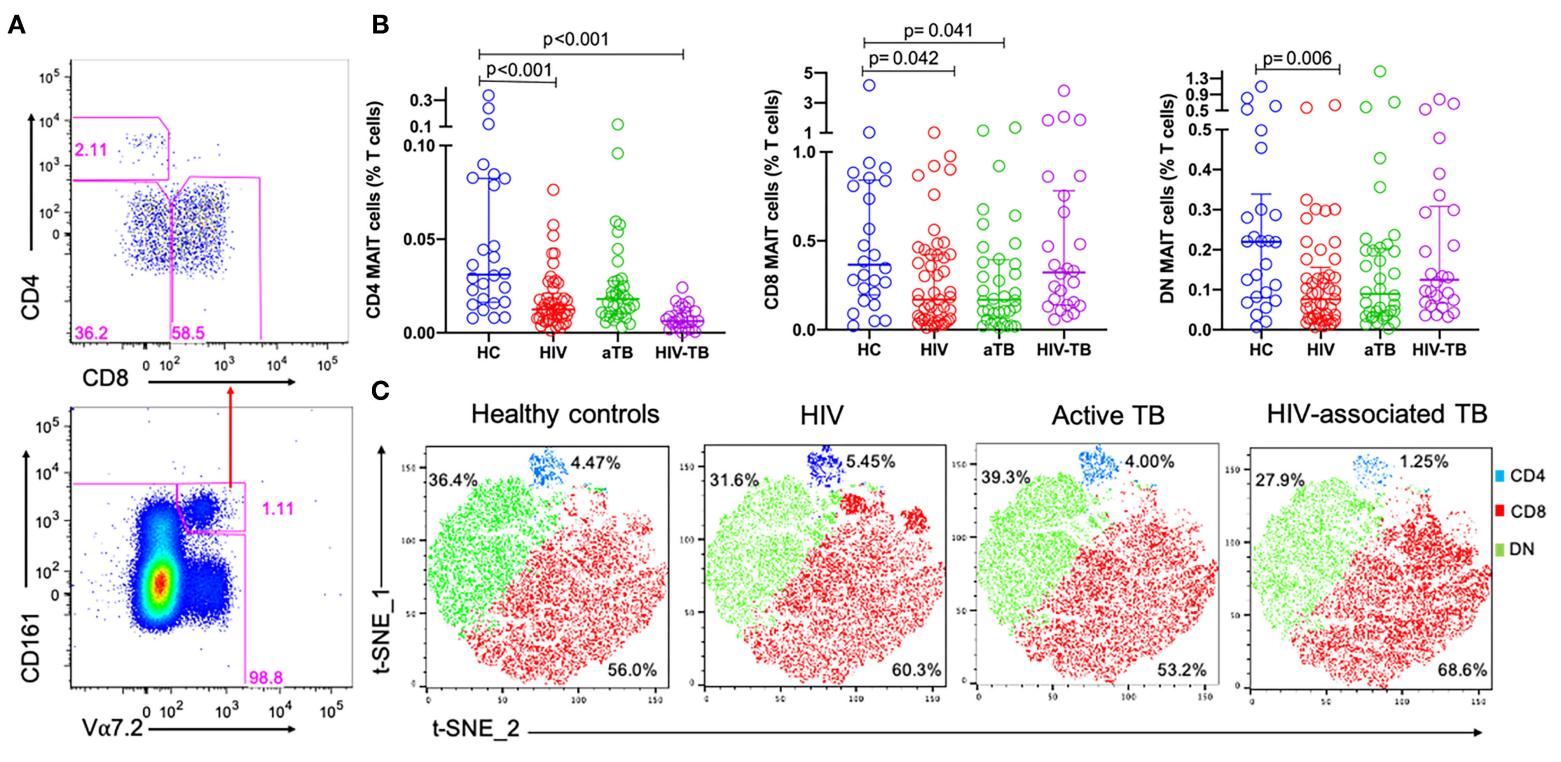
The frequencies of MAIT cell subsets during HIV infection, active TB, and HIV-associated TB. **(A)** Representative plots showing MAIT subsets gated from total MAIT cells. **(B)** Frequencies of CD4, CD8, and DN MAIT cell subsets in HC, HIV, aTB, and HIV-TB. **(C)** t-SNE analysis of MAIT cell subsets in the different study groups, CD4 MAIT- Blue, CD8 MAIT- Green, DN- Red. *P*-values reported from Kruskal-Wallis test with a Dunn's *post-hoc* test for multiple comparisons of groups and HC and *p* < 0.05 reported as statistically significant. HC, Healthy controls group; HIV, HIV only group; aTB, active TB only group; HIV-TB, HIV-associated TB group.

In order to evaluate any changes that occurred in frequencies at subset level, CD161^+^ Vα7.2^+^ MAIT cells were separated by CD4 and CD8 to obtain the three different MAIT cell subsets: CD4^+^CD8^−^ MAIT cells, CD8^+^CD4^−^ MAIT cells, and CD4^−^CD8^−^ MAIT cells, referred to as CD4, CD8 and DN MAIT cells, respectively (Figure 2A).

Figure 2B shows the frequencies of MAIT cell subsets as a proportion of total T cells. Frequencies of CD4 MAIT cells were lower in the groups with HIV (*p* < 0.001), and HIV-TB (*p* < 0.001) compared to CD4 MAIT cells in HC. CD8 MAIT cell frequencies were significantly lower in HIV (*p* = 0.042) and active TB groups (*p* = 0.041) compared to HC. DN MAIT cell frequencies were significantly lower in HIV group (*p* = 0.006) compared to HC (Figure 2B). From the t-SNE plots, it was clear that the CD8 (red) and DN (green) MAIT subsets make up significant proportions of MAIT cells compared to CD4 MAIT cells (blue). There were no large differences observed in the distributions of the different MAIT cell subsets except in the HIV-TB group where CD4 MAIT cells were substantially depleted (1.25%) compared to HC (4.47%) (Figure 2C).

Due to the disproportionate number of males and females in our HC compared to other groups, especially the active TB group, we adjusted for the effect of sex using ANCOVA and corrected for multiple comparisons using a Bonferroni test. Similar trends were observed in which frequencies of MAIT cells were significantly lower in HIV group compared to HC (*p* = 0.024). Frequencies of CD4 MAIT subsets were significantly lower in all groups compared to HC (HIV, *p* < 0.001; aTB, *p* = 0.016; HIV-TB- *p* < 0.001). There were no significant differences in the frequencies of CD8 MAIT subsets. Lastly, frequencies of DN MAIT subsets were lower in HIV only group compared to HC (*p* = 0.039). *P*-values before and after correction for sex differences are presented in Supplementary Table 1.

### MAIT Cells Are Functionally Impaired in Active TB and HIV-Associated TB

**Figure 3:**
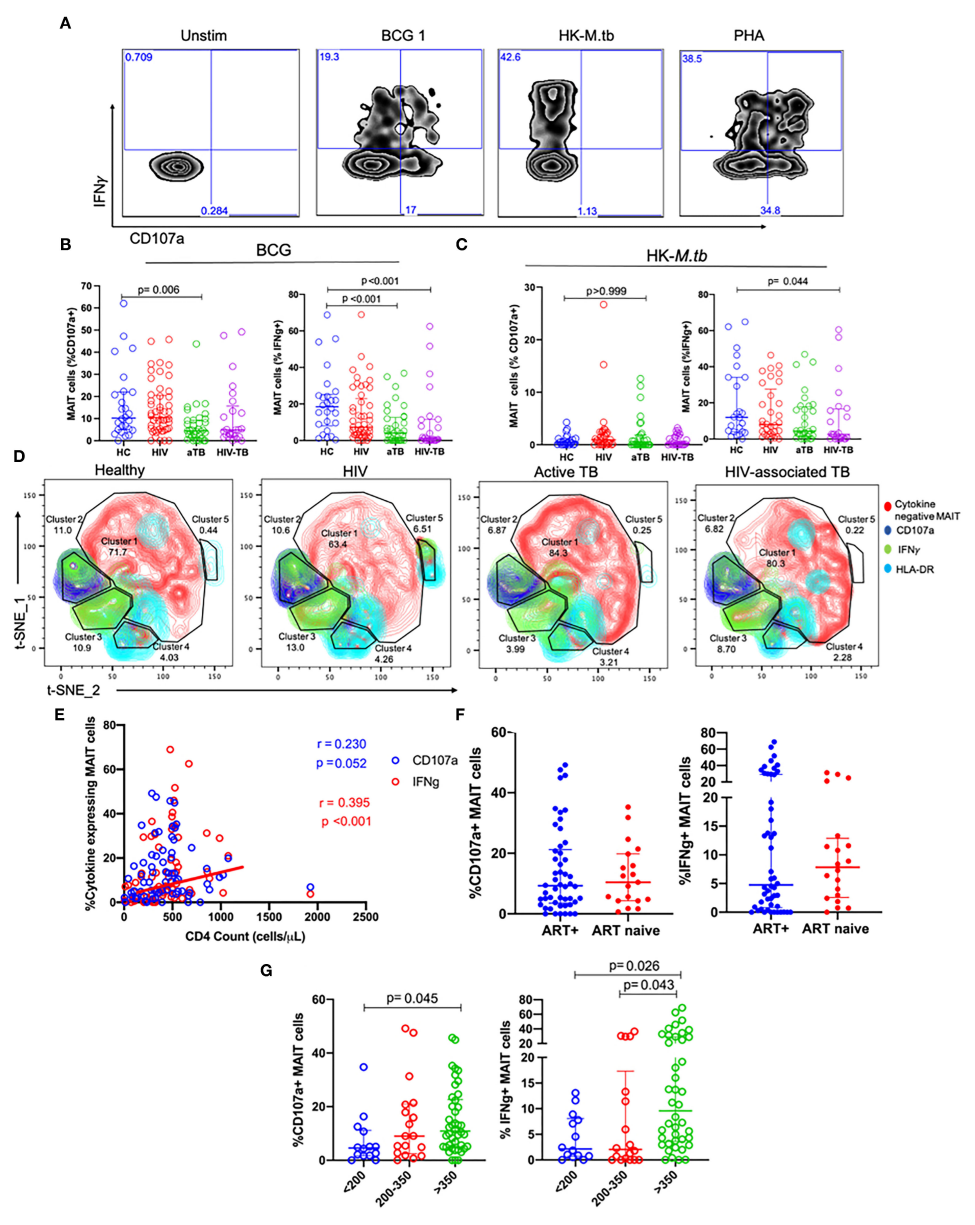
MAIT cell responses after BCG and HK-*M.tb* stimulation. **(A)** Representative plots showing IFNγ and CD107a expression after stimulation with BCG 1, heat-killed *M.tb* (HK-*M.tb*), and phytohemagglutinin (PHA). **(B,C)** Expression of CD107a and IFNγ after BCG stimulation and HK-*M.tb* stimulation, respectively. **(D)** Contour plots for t-SNE analysis of MAIT cell functions between the different groups. red-cytokine-MAIT cells; dark-blue-CD107a+ MAIT cells; green-IFNg+ MAIT cells; light blue-HLA-DR+ MAIT cells. **(E)** CD4 counts of HIV and HIV-TB group participants and frequencies of MAIT cells expressing CD107a and IFNγ, assessed using Spearman correlation. **(F)** Effect of ART on MAIT cells expressing CD107a and IFNγ. **(G)** Effect of CD4 counts on MAIT cells expressing CD107a and IFNγ. P-values reported from Kruskal-Wallis test with a Dunn's *post-hoc* test for multiple comparisons and *p* < 0.05 reported as statistically significant. HC, Healthy controls group; HIV, HIV only group; aTB, active TB only group; HIV-TB, HIV-associated TB group.

In order to assess MAIT cell functions, we evaluated the expression of CD107a (marker of degranulation), and IFNγ as functional markers of mycobacterial stimulation. Figure 3A shows the representative flow plots from the HC group for unstimulated cells and cells stimulated with BCG 1, HK*-M.tb*, and PHA.

In response to BCG 1 stimulation, no significant differences were observed in the frequencies of MAIT cells expressing CD107a between the HIV group and HC. Frequencies of MAIT cells expressing CD107a were significantly lower in the active TB group compared to HC (*p* = 0.006, Figure 3B). Frequencies of MAIT cells expressing IFNγ were lower in active TB group (*p* < 0.001) and HIV-associated TB (*p* < 0.001) compared to HC Figure 3B. Supplementary Table 2 shows the *p*-values and adjusted *p*-values for group comparisons of these MAIT frequencies and functions before and after correcting for multiple comparisons.

In response to HK-*M.tb* stimulation, we did not see any significant differences in the frequencies of MAIT cells expressing CD107a, although there were significantly lower frequencies of MAIT cells expressing IFNγ in people with HIV-TB compared to HC (*p* = 0.044) (Figure 3C). Upon stimulation with HK-*M.tb*, there were very low frequencies of MAIT cells expressing intracellular CD107a compared to BCG stimulation, as seen in Figures 3A,B.

We conducted t-SNE analyses on MAIT cells stimulated with BCG 1 (Figure 3D). In the t-SNE plots, a great proportion of MAIT cells were not responsive to BCG stimulation (cluster 1). The frequencies of MAIT cells expressing IFNγ were represented as green, MAIT cells expressing CD107a were blue and those expressing HLA-DR as cyan. The frequencies of MAIT cells expressing IFNγ were similar between HC and HIV group but were significantly lower in aTB compared to HC (6 vs. 19%). This depletion was coupled with the expansion in non-responsive MAIT cells in cluster 1. Finally, MAIT cells expressing IFNγ made up all of cluster 3, but a proportion of MAIT cells expressing CD107a also expressed IFNγ and these were found mainly in cluster 2.

Assessing the effect of HIV-related parameters on MAIT cell function, there was a positive, albeit weak correlation between frequencies of MAIT cells expressing IFNγ and the CD4 counts of participants with HIV (HIV and HIV-TB) (*p* < 0.001, *r* = 0.395) (Figure 3E). There were no significant differences in MAIT cells expressing CD107a, and IFNγ between people on ART and those not on ART (Figure 3F). Participants with CD4 counts above 350 cells/μl had higher frequencies of MAIT cells expressing CD107a than participants with CD4 counts below 200 cells/μl (*p* = 0.045) (Figure 3G). Similarly, there were higher frequencies of MAIT cells expressing IFNγ in participants with CD4 counts above 350 cells/μl than those with CD4 counts below 200 cells/μl (*p* = 0.021), and CD4 counts between 200 and 350 cells/μl (*p* = 0.043) (Figure 3G).

We also assessed the response to stimulation with live BCG using higher MOI (BCG 5). We found no significant differences in the frequencies of MAIT cells expressing CD107a in the HIV and HIV-TB group. There were lower frequencies of MAIT cells expressing CD107a in the aTB group (*p* = 0.015) compared to HC (Table 2). There were significantly lower frequencies of MAIT cells expressing IFNγ in the aTB (*p* = 0.006) and HIV-TB (*p* = 0.018) compared to HC (Table 2). *P*-values for groups comparisons before and after correcting for multiple comparisons are shown in Supplementary Table 3.

**Table 2:** Median and interquartile range (IQR) of frequencies of cytokine-expressing MAIT cells in response to stimulation with higher MOI BCG (BCG 5).

**Cytokine**	**HC**	**HIV**		**aTB**		**HIV-TB**	
	**Median (IQR)**	**Median (IQR)**	***p*-value**	**Median (IQR)**	***p*-value**	**Median (IQR)**	***p*-value**
CD107a	31.73 (19.59–58.17)	35.80 (20.65–47.29)	>0.999	20.41 (10.29–32.38)	0.015	30.80 (13.96–44.30)	0.694
HLA-DR	1.98 (1.00–5.00)	1.44 (0.01–4.55)	>0.999	0.71 (0.00–2.28)	0.105	1.62 (0.33–3.96)	>0.999
IFNγ	7.81 (2.54–17.52)	3.32 (0.62–9.72)	0.173	1.33 (0.03–5.08)	0.006	0.91 (0.03–7.87)	0.018

*Ns, Not significant; HC, Healthy controls; HIV, HIV group; aTB, active TB group; HIV-TB, HIV-associated TB group*.

Next, we looked at the functions of MAIT cell subsets in response to BCG 1 stimulation. For CD107a expression by MAIT cell subsets, there were no significant differences in the CD4 MAIT cell subset. There was lower expression of CD107a in the CD8 MAIT cell subset in aTB group (*p* = 0.003) and HIV-TB group (*p* = 0.026) compared to HC (Supplementary Figure 2A). Frequencies of DN MAIT cells expressing CD107a were significantly lower in the active TB group (*p* = 0.013) and HIV-TB group (*p* = 0.017) compared to HC (Supplementary Figure 2A).

There were lower frequencies of CD4 MAIT cells expressing IFNγ in HIV (*p* = 0.004), aTB (*p* = 0.003), and HIV-TB (*p* = 0.010) compared to HC (Supplementary Figure 2B). For CD8 MAIT cell subsets expressing IFNγ, frequencies were lower in aTB (*p* < 0.001), and HIV-TB (*p* < 0.001) compared to HC (Supplementary Figure 2B). Frequencies of DN MAIT subsets expressing IFNγ were significantly reduced in aTB (*p* = 0.001) and HIV-TB (*p* < 0.001) compared to HC (Supplementary Figure 2B). For the HIV group, frequencies of CD8, and DN MAIT cells that expressed IFNγ were similar to healthy controls.

After adjusting for sex differences, frequencies of MAIT cells expressing CD107a were lower in aTB group compared to HC (*p* = 0.046) and were not statistically significant in HIV and HIV-TB group. Frequencies of MAIT cells expressing IFNγ were lower in aTB (*p* = 0.024), but only a trend in HIV-TB group (*p* = 0.072) compared to HC (Supplementary Table 1). There were lower frequencies of CD8 MAIT subsets expressing IFNγ in the aTB group (*p* = 0.016) and HIV-TB (*p* = 0.045) compared to HC (Supplementary Table 1).

We also assessed the effects of the 24-h stimulation on the frequencies of MAIT cells. Representative flow plots in Supplementary Figure 3A show MAIT cell frequencies after 24-h. incubation in the unstimulated cells (top) and MAIT cell frequencies after 24-h. stimulation with BCG 1 (bottom). In each group, there were no significant differences in the frequencies of MAIT cells between unstimulated cells and cells stimulated with BCG 1 (Supplementary Figure 3B).

### MAIT Cells Are Significantly Activated in HIV Infection, Active TB, and in HIV-Associated TB

**Figure 4:**
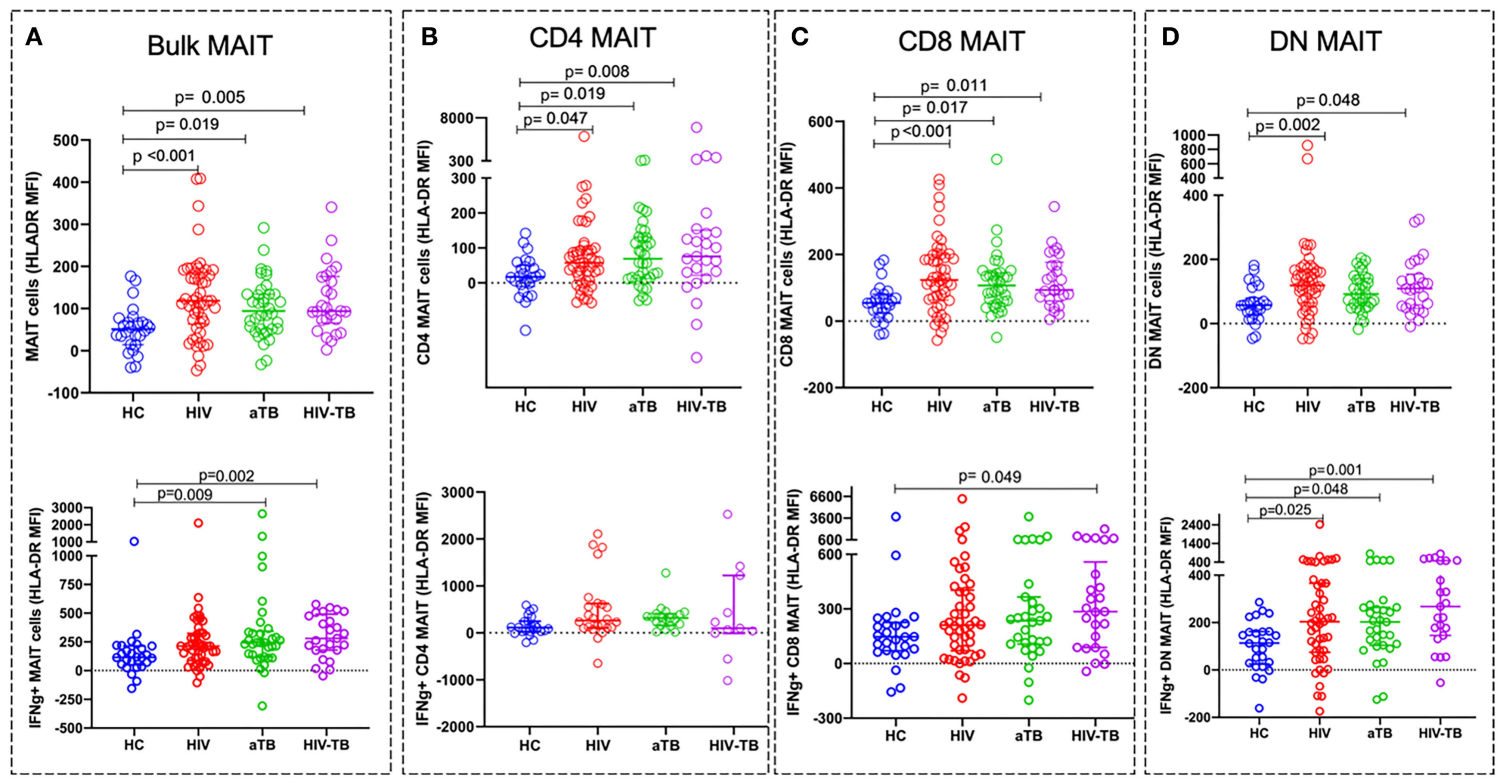
Activation status of MAIT cells in HIV, aTB, and HIV-associated TB represented by the MFI of HLA-DR. Activation status of resting (unstimulated) MAIT cells in HC, HIV, aTB group, and HIV-TB group (at the top) and activation status of BCG stimulated IFNγ+ MAIT cells and MAIT cell subsets (at the bottom). **(A)** Bulk MAIT cells. **(B)** CD4 MAIT subsets. **(C)** CD8 MAIT cells. **(D)** DN MAIT cells. *P*-values reported from Kruskal-Wallis test with a Dunn's *post-hoc* test for multiple comparisons and *p* < 0.05 reported as statistically significant. HC, Healthy controls group; HIV, HIV only group; aTB, active TB only group; HIV-TB, HIV-associated TB group.

We next investigated the effect of the different conditions on the activation status of MAIT cells. HLA-DR expression was used as a marker of T cell activation. There were no significant differences in frequencies of MAIT cells expressing HLA-DR between the groups (Supplementary Figure 4). HLA-DR median fluorescence intensity (MFI) on resting MAIT cells, was significantly higher in the HIV (*p* < 0.001), active TB (*p* = 0.019), and HIV-TB (*p* = 0.005) compared to HC (Figure 4A).

We also assessed specific MAIT cell activation status by analyzing the HLA-DR expression on MAIT cells expressing IFNγ following stimulation with BCG 1. We observed higher activation status in IFNγ ^+^ MAIT cells from active TB group (*p* = 0.009) and HIV-TB group (*p* = 0.002) compared to HC (Figure 4A).

For MAIT cell subset activation in unstimulated cells, we found that the MFI of HLA-DR on CD4 MAIT subsets was significantly higher in the HIV (*p* = 0.047), aTB (*p* = 0.019), and HIV-TB (*p* = 0.008) compared to HC (Figure 4B). CD8 subsets activation was also elevated in HIV (*p* < 0.001), aTB (*p* = 0.017), and HIV-TB (*p* = 0.011) compared to HC (Figure 4C). DN MAIT subset were found to be highly activated in HIV (*p* = 0.002), and HIV-TB (*p* = 0.048) (Figure 4D).

In response to BCG 1 stimulation, we observed that IFNγ^+^ CD8 MAIT cell subsets from HIV-TB had an elevated HLA-DR MFI (*p* = 0.049) compared to HC (Figure 4C). IFNγ^+^ DN MAIT subsets from HIV, aTB, and HIV-TB all had higher HLA-DR MFI than healthy controls (*p* = 0.025 in HIV, *p* = 0.048 in aTB, *p* = 0.001 in HIV-TB) (Figure 4D).

We assessed PD1 expression in a subset of HC (*n* = 10), HIV (*n* = 10), aTB (*n* = 10), and HIV-TB (*n* = 5) participants: the frequencies of MAIT cells expressing PD1 were significantly higher in the aTB group (*p* = 0.020) (Supplementary Figure 5) compared to HC. There were no differences in MAIT cell subset except for the DN where PD1 expression was lower in HIV-TB (*p* = 0.008) compared to HC.

After adjusting for sex differences, HLA-DR MFI on bulk MAIT cells were higher in HIV group (*p* < 0.001) and HIV-TB group (*p* = 0.030). There were no significant differences in HLA-DR MFI in CD4 MAIT cells. There were higher HLA-DR MFI in CD8 MAIT cells and DN MAIT cells from HIV group (CD8 MAIT subset, *p* = 0.002; DN MAIT subset, *p* = 0.015) compared to HC (Supplementary Table 1).

### MAIT Cell Functions but Not Frequencies Are Restored After 10 Weeks of TB Treatment

**Figure 5:**
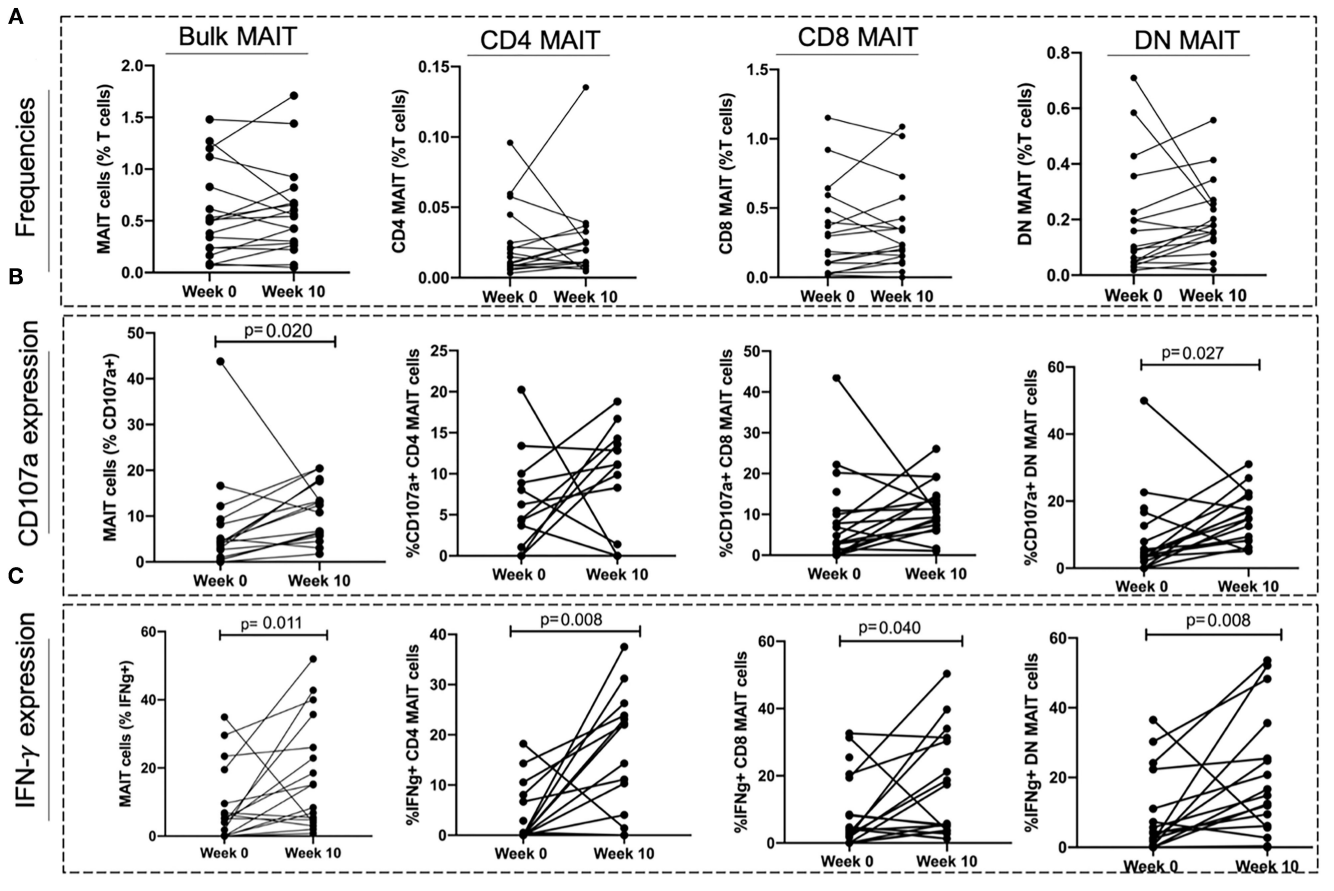
Effect of TB treatment on MAIT cell frequencies and functions. **(A)** Frequencies of MAIT cells and MAIT cell subsets at start of treatment (Week 0) and at 10 weeks of TB treatment (Week 10). **(B,C)** MAIT cell and MAIT cell subset responses (CD107a expression and IFNγ expression) at start of treatment (Week 0) and after 10 weeks of TB treatment (Week 10). *P*-values reported from Wilcoxon-ranked test between paired samples and *p* < 0.05 reported as statistically significant.

To investigate whether TB treatment induced any changes in MAIT cell frequencies and functions, a subset of 18 individuals from the aTB group were sampled after 6–10 (median of 8) weeks of TB treatment. No significant differences were observed in the MAIT cell frequencies between baseline (week 0) and week 10 of TB treatment (Figure 5A). There was an increase in the frequencies of MAIT cells expressing CD107a (*p* = 0.020), and IFNγ (*p* = 0.011) after 10 weeks of TB treatment (Figures 5B,C).

We found no changes in CD4 and CD8 MAIT cells expressing CD107a after TB treatment (Figure 5B). The frequencies of DN MAIT cells expressing CD107a were higher after 10 weeks of TB treatment (*p* = 0.027) (Figure 5B). Frequencies of all MAIT cell subsets expressing IFNγ were higher after 10 weeks treatment (IFNγ^+^ CD4 MAIT; *p* = 0.008, IFNγ^+^ CD8 MAIT; *p* = 0.040, IFNγ^+^ DN MAIT; *p* = 0.008) (Figure 5C).

## Discussion

We investigated both the frequencies and function of MAIT cells in healthy adults, adults with HIV, active TB, and HIV-associated TB in a high endemic TB setting in South Africa. MAIT cell frequencies were depleted in people with HIV infection, and with active TB. Although MAIT cells in HIV infected, active TB, and HIV-associated TB patients had an elevated activation status, their functions were retained in response to BCG stimulation in HIV infected patients. In patients with active TB, MAIT cells were functionally impaired with lower expression of IFNγ and increased activation. There were also increased frequencies of MAIT cells expressing PD1 in active TB. We observed that MAIT cell frequencies did not recover after 10 weeks of TB treatment, but their functional capacity (measured by CD107a and IFNγ expression) did increase.

The depletion of blood MAIT cell frequencies in people with HIV and active TB has been described previously, and our findings are consistent with these previous studies (21–23), with the exception of the study by Suliman et al. (26). Suliman et al. (26) defined MAIT cells as CD3+ 5-OP-RU MR1 tetramer+, and showed that in a cohort of TB patients in South Africa and Peru that there was no significant difference in MAIT cell frequencies between TB patients and healthy controls, both with and without latent TB infection. We observed a non-significant decrease in MAIT cell frequencies in people with active TB when MAIT cells were defined using antibodies, but this decrease was significant when MAIT cells were more precisely defined using tetramers. It is thought that depletion of MAIT cells in blood during infection may be due to their recruitment to the sites of infection (23, 27, 28). Leeansyah and colleagues reported that although MAIT cells were lost in the blood in HIV-1 infection, their frequencies were preserved in the rectal mucosa, suggesting that they are either preserved in these sites or that they migrate to mucosal sites as a response to infection and to maintain mucosal integrity (23, 25). More recently, it has been shown that there were increased MAIT cell frequencies in gut mucosa of people in the early stages of HIV infection compared to healthy controls suggesting early recruitment to mucosal tissues (29).

In TB disease, evidence also suggests that MAIT cells might be recruited to sites of infection and have been shown to be lost in blood of people with active TB but enriched in the lungs, with a capacity to secrete IFNγ in response to co-culture with *M.tb*-infected lung epithelial cells (9). More recently, Wong et al. (28) showed that although individuals with active TB had fewer circulating TRAV1-2^+^ CD8 T cells, they had 3 times more TRAV1-2^+^ CD8^+^ T cells in the bronchoalveolar lavage fluid (BAL) compared to matched healthy controls, suggesting recruitment to the lungs.

Interestingly, whereas we found lower frequencies in HIV infection and active TB, we did not see any significant differences in HIV-associated TB. Since participants with HIV-associated TB had lower CD4 counts and higher HIV viral loads, this observation could be explained by the severe depletion of non-MAIT T cells in the CD3 compartment which leads to the relative enrichment of the MAIT compartment. Saeidi et al. (30) did not observe any significant differences in the frequencies of MAIT cells between people that had HIV-associated TB who were on ART and healthy controls. They showed that a group of HIV-associated TB patients who were not on ART had lower frequencies than healthy controls. This suggests that ART may have partially restored MAIT cell frequencies in HIV-associated TB. It is worth noting though, that the study defined MAIT cells as CD161^++^ CD8^+^ T cells whereas we defined them as CD161^+^Vα7.2^+^ T cells. Another difference between the studies is the sample size; each of their groups had 10 individuals, fewer than in our study.

Similar to previous studies (22, 23), we did not see any differences in MAIT cell frequencies in individuals on ART and those not on ART, and we also did not see any correlations between MAIT cell frequencies and HIV VL. The observed inverse correlation of MAIT cell frequencies with CD4 counts seems largely driven by individuals with HIV-TB since MAIT cells in this group were inversely correlated with CD4 counts but this was not evident in the HIV only group.

The results from MR1-tetramer defined MAIT cells showed significantly lower frequencies of MAIT cells in active TB than healthy controls but not in HIV group compared to healthy controls. This was different from the results from Vα7.2 identified MAIT cells which showed lower MAIT cells in HIV. It is not clear if this observation is due to the lower sample size. It could suggest that antibody staining may be technically insufficient for detecting all MAIT cells, especially if these markers are regulated differentially under different disease states. Gherardin et al. have suggested that this could lead to some MAIT cells being missed due to expression of alternate T cell receptors and low expression of CD161 (14, 31, 32). Some cells can be misclassified as MAIT cells and it has been demonstrated that some CD161^+^Vα7.2^+^ MAIT cells were unable to bind to MR1-loaded tetramers (14, 33), and Swarbrick et al. (14) suggested that these cells share similar transcriptional profiles with MR1-restricted T cells but need further functional characterization.

We investigated MAIT cell functions by assessing the expression of CD107a and IFNγ in response to BCG and HK-*M.tb*. In response to BCG, we observed no significant differences in the frequencies of MAIT cells expressing IFNγ and CD107a between the HIV group and healthy controls. This was in contrast to previous studies that showed that HIV positive individuals had fewer MAIT cells expressing IFNγ, CD107a, and granzyme B compared to healthy controls (23, 34). One fundamental difference between our study and these previous studies is that they both assessed MAIT cell responses to fixed *Escherichia coli* stimulation whereas we assessed functions using live BCG. Our findings were similar to those studies that did not report any significant differences in the MAIT cell functions between healthy controls and HIV positive individuals (25, 29). However, both of those studies investigated MAIT cell responses to MAIT cell ligand 5-OP-RU, IL-12, and IL-18 stimulation, and in early HIV infection where these functions may be maintained. Stimulation with HK-*M.tb* yielded very low expression of CD107a on MAIT cells and this is likely due to the denaturing of MAIT cell activating ligand due to heat-killing which may have led to an inability to activate MAIT cells in an MR1-dependent manner. However, there were increased IFNγ responses which indicate that HK-*M.tb* may have still been able to activate MAIT cells through MR1-independent mechanisms. Interestingly, we only saw reduced frequencies of MAIT cells expressing IFNγ in HIV-TB and not in aTB, suggesting that *M.tb* specific responses were still maintained in HIV and aTB group but not in HIV-TB. Although the specific MAIT cell ligands in both BCG and HK-*M.tb* have not been described, the observed MAIT cell responses to BCG in our study may be through both the MR1-dependent and MR1-independent mechanisms.

In TB participants, we saw that there were lower frequencies of MAIT cells expressing CD107a and IFNγ. These results were consistent with those previously reported (9, 21). Interestingly, Wong et al. (28) recently showed that TRAV1-2^+^ CD8 T cells (MAIT cells) in bronchoalveolar lavage (BAL) fluid had greater functional responses (TNF-α expression) to *Mycobacterium smegmatis*-infected APCs than peripheral blood MAIT cells in patients with untreated active TB. This suggests that there is functional impairment of MAIT cells during TB disease, but this impairment may not be in all compartments. This observed dysfunction has also been described in other T cell subsets and has been attributed to T cell exhaustion which is driven by persistent antigen exposure and microbial translocation in HIV-associated TB (35).

It is possible that this diminished functional capacity is due to inhibition resulting from increased expression of inhibitory receptors such as PD1 and TIM-3 (34–36). We investigated whether PD1 expression could be a contributing factor as previously described (21, 30, 37). We found that resting MAIT cells in our disease groups were significantly more activated than healthy controls, and that in TB disease (active TB and HIV-associated TB) MAIT cells expressing IFNγ following mycobacterial stimulation, were more activated than healthy controls. This suggests that there was already immune activation *in vivo*. We also observed that there were more MAIT cells expressing PD1 in active TB, which could indicate that there is immune inhibition *via* PD1 expression as a regulatory mechanism in chronic activation, which can lead to cell death (38, 39). Jiang et al. (21) have demonstrated that this impairment in MAIT cell function can be rescued by anti-PD1 therapy.

T cell functional impairment has also been associated with mycobacterial load as Day et al. (40) demonstrated that T cell functionality worsened with higher mycobacterial load and recovers after 6 months of TB treatment. We have also observed that the functional capacity in MAIT cells improves after 6–10 weeks of TB treatment, suggesting that we are seeing an improvement in MAIT cell functions as bacterial load decreases. Similar to bulk MAIT cells, MAIT cell subset frequencies were not restored after the first 10 weeks of TB treatment, but their functions were restored.

Our findings suggest that HIV positive participants with higher CD4 counts had more functional MAIT cells than those with lower CD4 counts, and the HIV VL and ART status did not seem to impact MAIT cell functions, which is in contrast to Leeansyah et al. (23) who have reported increased frequencies of MAIT cells expressing IFNγ in people on combination ART (cART). However, in their cohort, the analyses were conducted on paired samples from people before and after cART.

It is important to note that our healthy controls (and participants with HIV only) were predominantly latently infected with TB, thus our comparisons would most likely be representative of latent TB infection vs. active TB. One of the limitations of our study is that we did not collect any BAL samples and samples from other sites of infection and thus can only postulate that the observed reductions are due to recruitment to lungs and other sites of infection. Our HC and HIV groups had larger proportions of females than males, and our aTB group predominantly consisted of males, we adjusted for sex differences using ANCOVA and we found that sex did not significantly change our observed results. Another limitation of our study was the relatively small sample size in the different participant groups. A strength of our study was that the participant groups were well-characterized in terms of TB diagnosis and HIV and ART status. To our knowledge, this is the first study to investigate MAIT cell frequencies and functions in TB, HIV, and HIV-associated TB in South Africa.

In conclusion, we found that MAIT cells are reduced in blood of people with HIV and active TB but MAIT cell functions are maintained in HIV and impaired in active TB and HIV-associated TB with significant activation in HIV, active TB, and HIV-associated TB. We have shown that MAIT cell frequencies were not restored after 10 weeks of TB treatment, but functional capacity did improve. These findings and those from previous studies suggest that MAIT cells play a role in TB pathogenesis, the precise nature of which remains to be defined. The alterations in MAIT cell frequencies and functions we observed may reduce the capacity of MAIT cells to play a protective role in the immune response to these two pathogens.

## Data Availability Statement

The raw data supporting the conclusions of this article will be made available by the authors, without undue reservation.

## Ethics Statement

The studies involving human participants were reviewed and approved by University of Cape Town Faculty of Health Sciences Human Research Ethics Committee, HREC REF 011/2017. The patients/participants provided their written informed consent to participate in this study.

## Author Contributions

MS conceived and supervised all aspects of the study. AB performed all experiments and analyses. GM co-conceived the study and provided intellectual inputs. KW, DML, and DAL provided intellectual input on study design, experimental set-up, and analyses. CS, RG, BS, SS, J-PK, AW, and RN provided clinical guidance and recruited study participants. AG provided intellectual and technical input on data analyses. All authors read and approved the final version of the manuscript.

## Conflict of Interest

The authors declare that the research was conducted in the absence of any commercial or financial relationships that could be construed as a potential conflict of interest.

## Supplementary Material

Supplementary Figure 1 Frequencies of MAIT cells using MR-1 tetramer. **(A)** Representative flow plots showing CD4 and CD8 T cells in the different study groups. **(B)** Representative flow plots showing MAIT cells identified with (i) 5-OP-RU MR1 tetramer and (ii) 6-FP MR1 loaded control tetramer. **(C)** Frequencies of MAIT cells in healthy controls, HIV group, aTB group, and HIV-associated TB group. **(D)** Correlation between antibody defined and MR1-OPRU tetramer defined MAIT cells. **(E)** Flow plots comparing frequencies of Vα7.2 antibody stained MAIT cells (top) and MR1 5-OP-RU stained MAIT cells (bottom). **(F)** Comparisons of MAIT cell frequencies in MAIT cell identified with Vα7.2 antibody and MAIT cells identified with MR1 5-OP-RU tetramer.

Supplementary Figure 2 CD4, CD8, and DN MAIT cell subset responses to BCG 1 stimulation. **(A)** Expression of CD107a and **(B)** IFNγ in the different MAIT cell subsets in response to BCG 1 stimulation. *P*-values reported from Kruskal-Wallis test with a Dunn's *post-hoc* test for multiple comparisons and *p* < 0.05 reported as statistically significant. HC, Healthy controls group; HIV, HIV only group; aTB, active TB only group; HIV-TB, HIV-associated TB group.

Supplementary Figure 3 The effect of 24 h stimulation of the frequencies of MAIT cells. **(A)** Representative flow plots showing MAIT cell frequencies in unstimulated cells (top) and BCG stimulated cells (bottom). **(B)** Frequencies of MAIT cells in each group before and after BCG stimulation. *P*-value represents *p*-value from Wilcoxon rank test between paired samples.

Supplementary Figure 4 Frequencies of MAIT cells expressing HLA-DR, a marker of T cell activation. **(A)** Representative flow plot showing frequencies of HLA-DR MAIT cells. **(B)** Summary plots showing the frequencies of MAIT cells expressing HLA-DR. **(C)** Summary plots of HLA-DR expression in the CD4, CD8, and DN MAIT cell subsets.

Supplementary Figure 5 Expression of PD1 by resting MAIT cells and MAIT cell subsets during HIV, active TB, and HIV-associated TB. **(A)** Representative flow plots showing PD1 expression on MAIT cells. **(B)** PD1 expression on MAIT cells. **(C)** %PD1 expression on CD4 MAIT subset. **(D)** %PD1 expression on CD8 MAIT cell subset. **(E)** %PD1 expression on DN MAIT subset. *P*-values reported from Kruskal-Wallis test with a Dunn's *post-hoc* test for multiple comparisons and *p* < 0.05 reported as statistically significant. HC, Healthy controls group; HIV, HIV only group; aTB, active TB only group; HIV-TB, HIV-associated TB group.

Supplementary Table 1 Significance levels for group comparisons for bulk MAIT cell and MAIT cell subset frequencies, functions, and activation before and after adjusting for the effect of gender using Analysis of Covariance (ANCOVA) and Bonferroni correction for multiple comparisons.

Supplementary Table 2 p-values for group comparisons before and after adjusting for multiple comparisons using Dunn's test.

Supplementary Table 3 p-values of group comparisons for BCG 5 stimulated MAIT cells, before and after adjusting for multiple comparisons using Dunn's test.
