# Supplementary figures and images for: Functional and Activation Profiles of Mucosal-Associated Invariant T Cells in Patients With Tuberculosis and HIV in a High Endemic Setting
Source: Front Immunol. 2021 Mar 22;12:648216. doi: 10.3389/fimmu.2021.648216 (PMC8019701; doi:10.3389/fimmu.2021.648216)

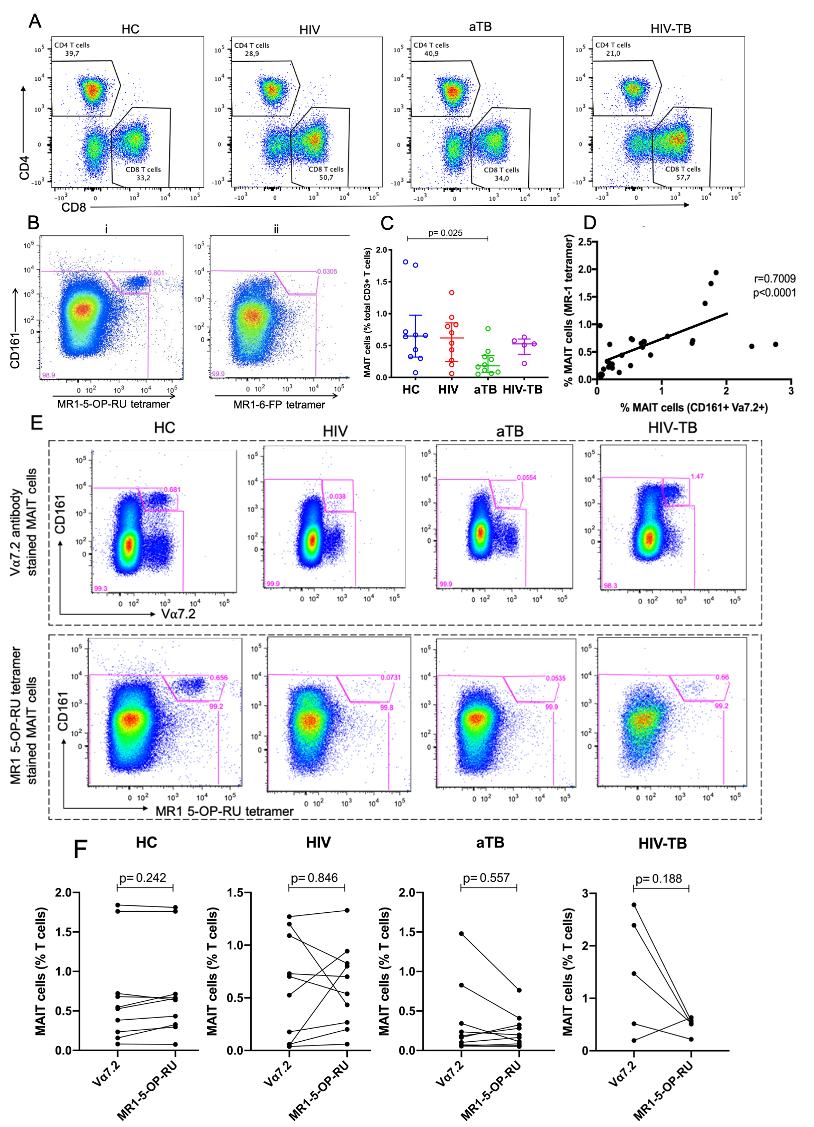

Supplement: Supplementary Figure 1 — Frequencies of MAIT cells using MR-1 tetramer. (A) Representative flow plots showing CD4 and CD8 T cells in the different study groups. (B) Representative flow plots showing MAIT cells identified with (i) 5-OP-RU MR1 tetramer and (ii) 6-FP MR1 loaded control tetramer. (C) Frequencies of MAIT cells in healthy controls, HIV group, aTB group, and HIV-associated TB group. (D) Correlation between antibody defined and MR1-OPRU tetramer defined MAIT cells. (E) Flow plots comparing frequencies of Vα7.2 antibody stained MAIT cells (top) and MR1 5-OP-RU stained MAIT cells (bottom). (F) Comparisons of MAIT cell frequencies in MAIT cell identified with Vα7.2 antibody and MAIT cells identified with MR1 5-OP-RU tetramer. [file Image_1.JPEG]

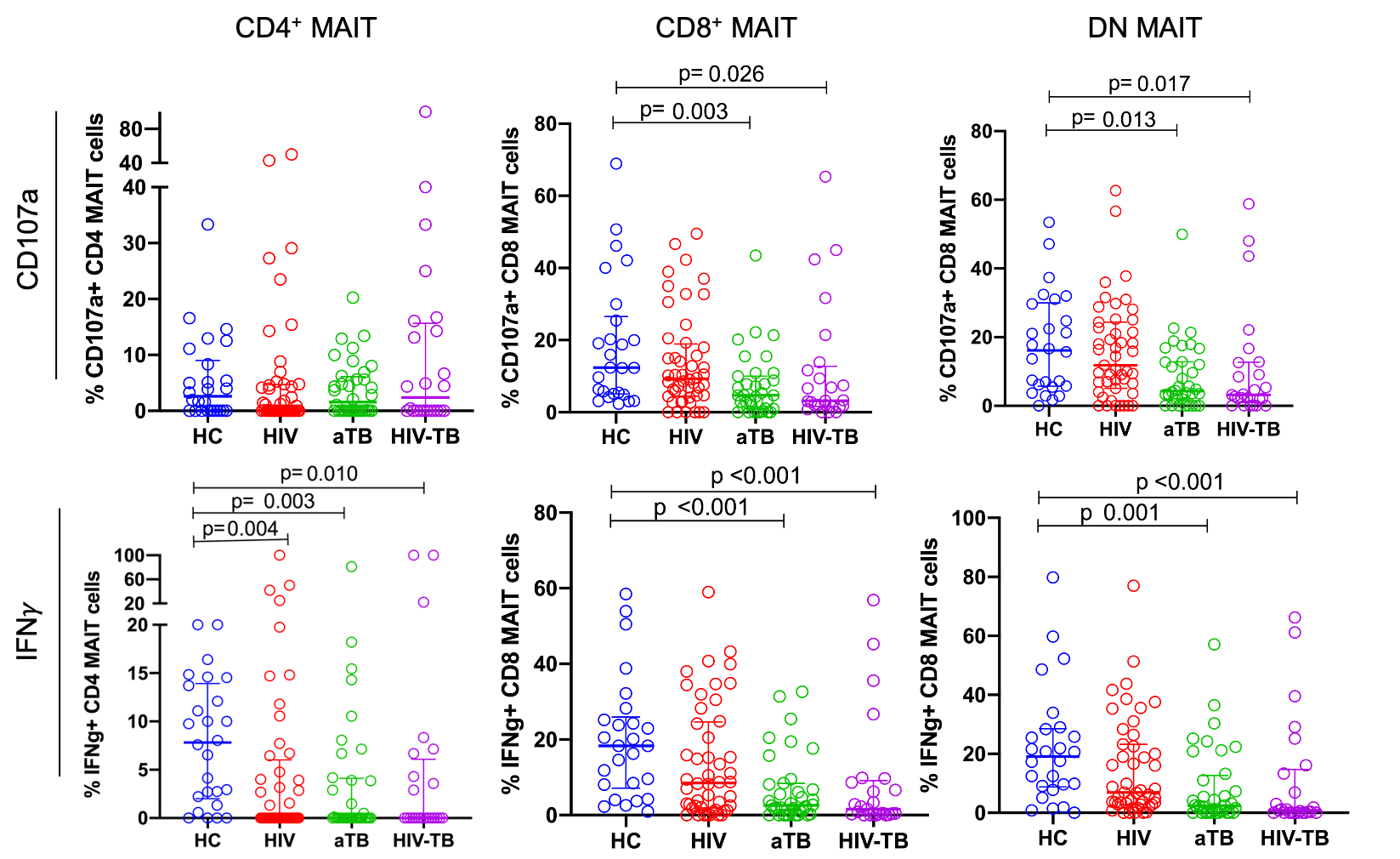

Supplement: Supplementary Figure 2 — CD4, CD8, and DN MAIT cell subset responses to BCG 1 stimulation. (A) Expression of CD107a and (B) IFNγ in the different MAIT cell subsets in response to BCG 1 stimulation. P-values reported from Kruskal-Wallis test with a Dunn's post-hoc test for multiple comparisons and p < 0.05 reported as statistically significant. HC, Healthy controls group; HIV, HIV only group; aTB, active TB only group; HIV-TB, HIV-associated TB group. [file Image_2.JPEG]

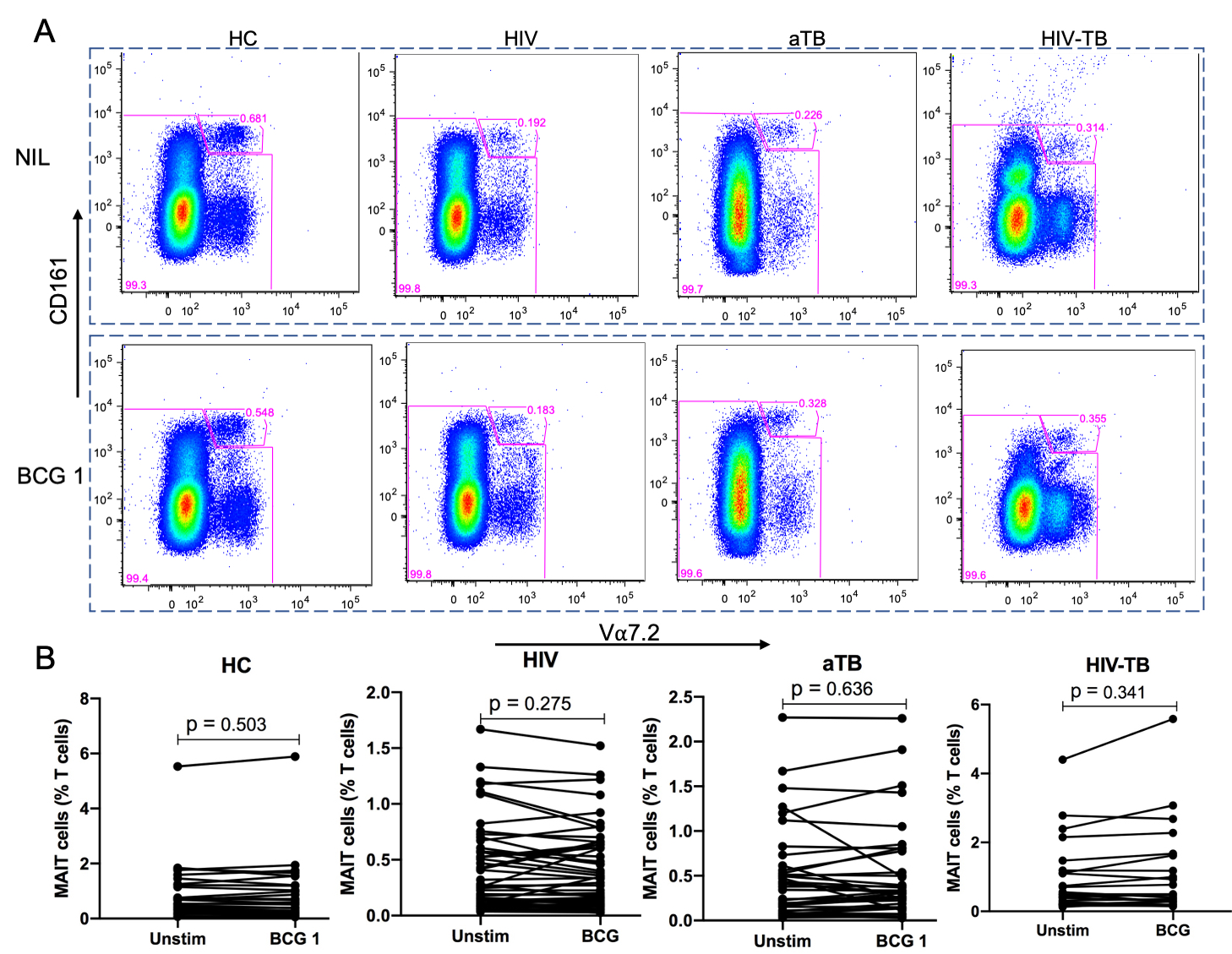

Supplement: Supplementary Figure 3 — The effect of 24 h stimulation of the frequencies of MAIT cells. (A) Representative flow plots showing MAIT cell frequencies in unstimulated cells (top) and BCG stimulated cells (bottom). (B) Frequencies of MAIT cells in each group before and after BCG stimulation. P-value represents p-value from Wilcoxon rank test between paired samples. [file Image_3.JPEG]

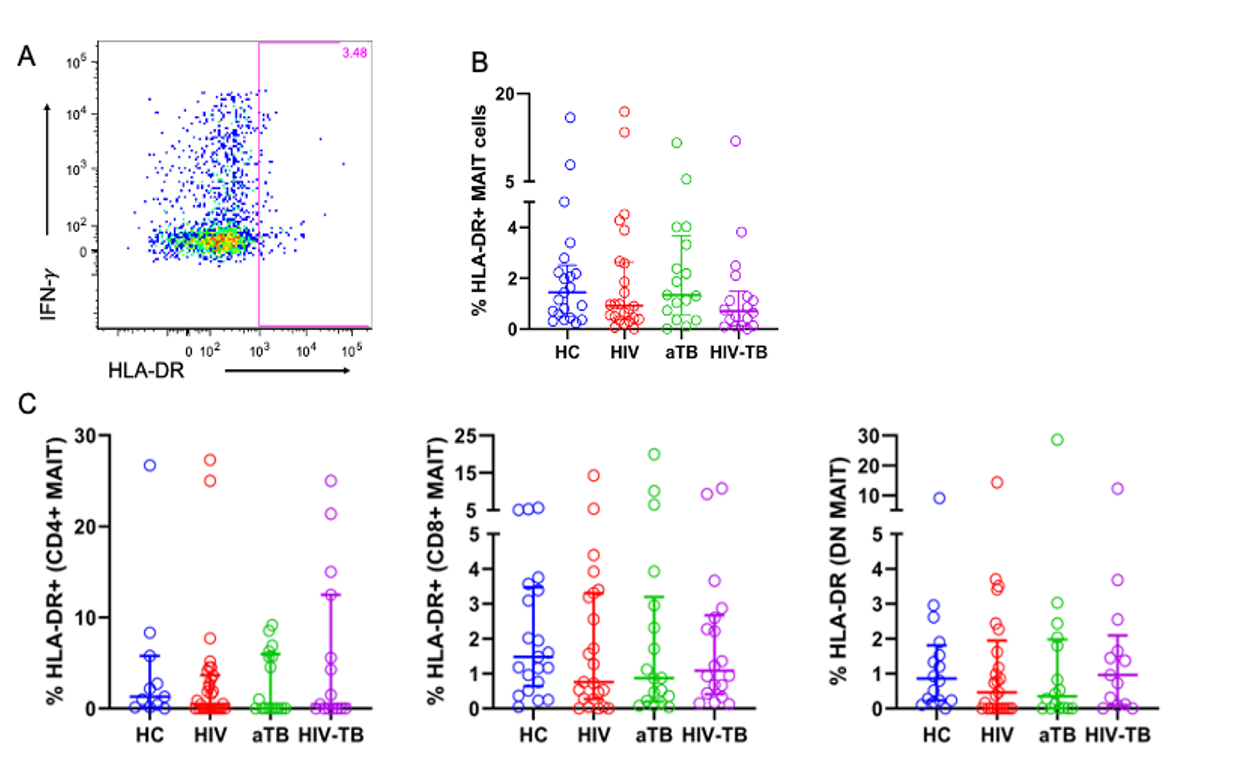

Supplement: Supplementary Figure 4 — Frequencies of MAIT cells expressing HLA-DR, a marker of T cell activation. (A) Representative flow plot showing frequencies of HLA-DR MAIT cells. (B) Summary plots showing the frequencies of MAIT cells expressing HLA-DR. (C) Summary plots of HLA-DR expression in the CD4, CD8, and DN MAIT cell subsets. [file Image_4.JPEG]

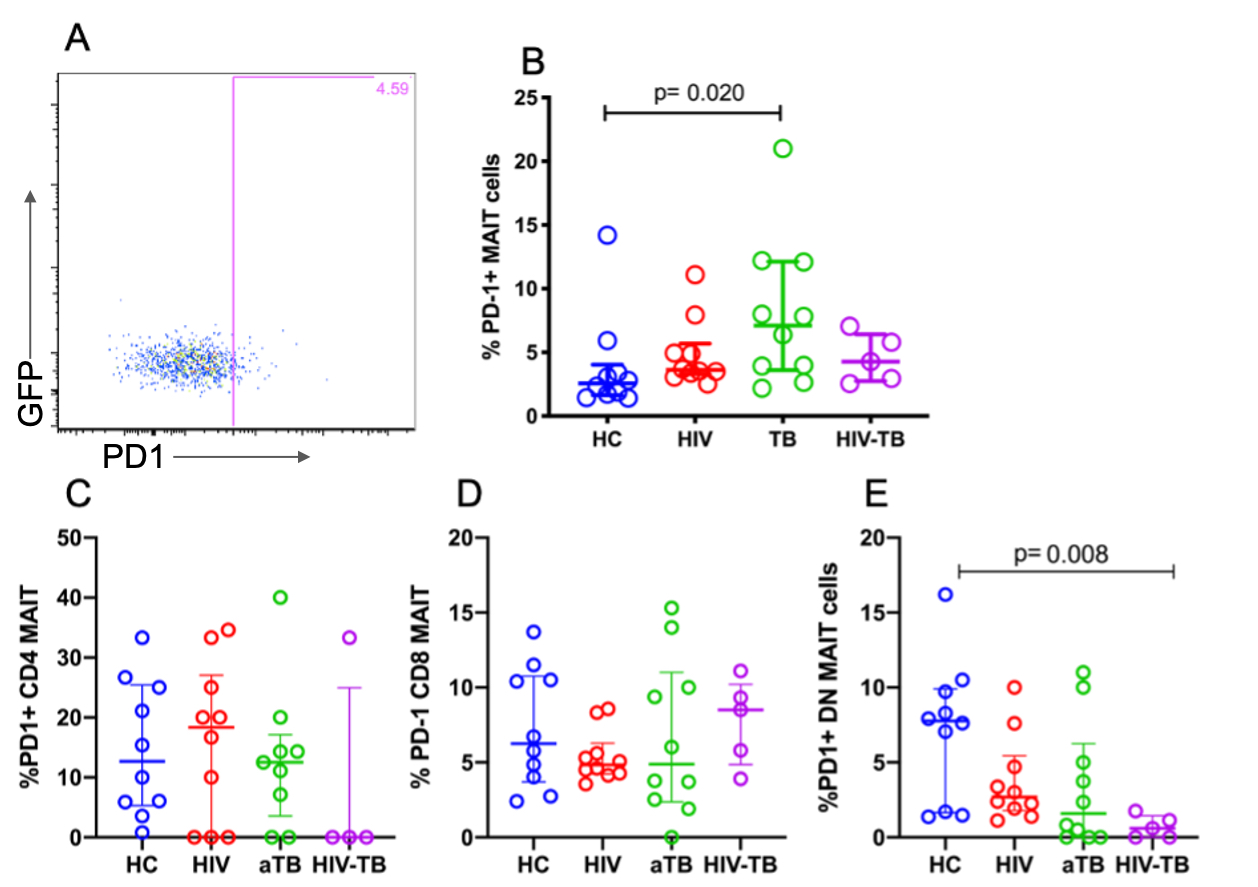

Supplement: Supplementary Figure 5 — Expression of PD1 by resting MAIT cells and MAIT cell subsets during HIV, active TB, and HIV-associated TB. (A) Representative flow plots showing PD1 expression on MAIT cells. (B) PD1 expression on MAIT cells. (C) %PD1 expression on CD4 MAIT subset. (D) %PD1 expression on CD8 MAIT cell subset. (E) %PD1 expression on DN MAIT subset. P-values reported from Kruskal-Wallis test with a Dunn's post-hoc test for multiple comparisons and p < 0.05 reported as statistically significant. HC, Healthy controls group; HIV, HIV only group; aTB, active TB only group; HIV-TB, HIV-associated TB group. [file Image_5.JPEG]
