# Supplementary material for: Functional and Activation Profiles of Mucosal-Associated Invariant T Cells in Patients With Tuberculosis and HIV in a High Endemic Setting
Source: Front Immunol. 2021 Mar 22;12:648216. doi: 10.3389/fimmu.2021.648216 (PMC8019701; doi:10.3389/fimmu.2021.648216)
Supplement: Supplementary Table 1 — Significance levels for group comparisons for bulk MAIT cell and MAIT cell subset frequencies, functions, and activation before and after adjusting for the effect of gender using Analysis of Covariance (ANCOVA) and Bonferroni correction for multiple comparisons. [file Table_1.docx]

**Supplementary Table 1: Significance levels for group comparisons for bulk MAIT cell and MAIT cell subset frequencies, functions, and activation before and after adjusting for the effect of gender using Analysis of Covariance (ANCOVA) and Bonferroni correction for multiple comparisons.**

|  |  | **HIV** | | **aTB** | | **HIV-TB** | |
| --- | --- | --- | --- | --- | --- | --- | --- |
|  |  | **p-value^a^** | **p-value^b^** | **p-value^a^** | **p-value^b^** | **p-value^a^** | **p-value^b^** |
| **Frequencies (%T cells)** | MAIT cells | **0.009** | **0.024** | 0.057 | 0.235 | >0.999 | 1.000 |
|  | CD4 MAIT cells | **<0.001** | **<0.001** | 0.068 | **0.016** | **<0.001** | **<0.001** |
|  | CD8 MAIT cells | **0.042** | 0.142 | **0.041** | 0.434 | >0.999 | 1.000 |
|  | DN MAIT cells | **0.006** | **0.039** | 0.085 | 0.732 | >0.999 | 1.000 |
| **CD107a expression**  **(%CD107a+)** | MAIT cells | >0.999 | 1.000 | **0.006** | **0.046** | 0.191 | 0.895 |
|  | CD4 MAIT cells | 0.910 | 1.000 | >0.999 | 1.000 | >0.999 | 0.422 |
|  | CD8 MAIT cells | 0.753 | 0.938 | **0.003** | 0.058 | **0.026** | 0.542 |
|  | DN MAIT cells | 0.999 | 1.000 | **0.013** | 0.097 | **0.017** | 0.370 |
| **IFN**γ  **Expression**  **(%IFN**γ+) | MAIT cells | 0.313 | 0.377 | **<0.001** | **0.024** | **<0.001** | 0.072 |
|  | CD4 MAIT cells | **0.004** | 1.000 | **0.003** | 1.000 | **0.010** | 1.000 |
|  | CD8 MAIT cells | 0.117 | 0.424 | **<0.001** | **0.016** | **<0.001** | **0.045** |
|  | DN MAIT cells | 0.381 | 0.538 | **<0.001** | 0.057 | **<0.001** | 0.142 |
| **HLA-DR MFI** | MAIT cells | **<0.001** | **<0.001** | **0.019** | 0.342 | **0.005** | **0.030** |
|  | CD4 MAIT cells | **0.047** | 1.000 | **0.019** | 1.000 | **0.008** | 0.353 |
|  | CD8 MAIT cells | **<0.001** | **0.002** | **0.017** | 0.287 | **0.011** | 0.106 |
|  | DN MAIT cells | **0.002** | **0.015** | 0.062 | 1.000 | **0.048** | 0.378 |

1. p-values after Kruskal-Wallis test and a Dunns test to correct for multiple comparisons.
2. p-values after adjusting for the effect of sex using an ANCOVA and a Bonferroni test to correct for multiple comparisons.
