# Supplementary material for: Functional and Activation Profiles of Mucosal-Associated Invariant T Cells in Patients With Tuberculosis and HIV in a High Endemic Setting
Source: Front Immunol. 2021 Mar 22;12:648216. doi: 10.3389/fimmu.2021.648216 (PMC8019701; doi:10.3389/fimmu.2021.648216)
Supplement: Supplementary Table 2 — p-values for group comparisons before and after adjusting for multiple comparisons using Dunn's test. [file Table_2.docx]

**Supplementary Table 2: p-values for group comparisons before and after adjusting for multiple comparisons using Dunn’s test.**

|  | | **HIV** | | **aTB** | | **HIV-TB** | |
| --- | --- | --- | --- | --- | --- | --- | --- |
|  |  | **p-value** | **adjusted p-value** | **p-value** | **adjusted p-value** | **p-value** | **adjusted p-value** |
| **Frequencies** | **Bulk MAIT** | **0.003** | **0.009** | **0.020** | 0.057 | 0.793 | >0.999 |
|  | **CD4 subset** | **<0.001** | **<0.001** | **0.008** | 0.068 | **<0.001** | **0.001** |
|  | **CD8 subset** | **0.016** | **0.042** | **0.016** | **0.041** | 0.723 | >0.999 |
|  | **DN subset** | **0.003** | **0.006** | **0.026** | 0.085 | **0.002** | >0.999 |
| **HLA-DR MFI** | **Bulk MAIT** | **<0.001** | **<0.001** | **0.003** | **0.019** | **<0.001** | **0.005** |
|  | **CD4 subset** | **0.011** | **0.047** | **0.007** | **0.019** | **0.004** | **0.008** |
|  | **CD8 subset** | **<0.001** | **<0.001** | **0.003** | **0.017** | **0.002** | **0.011** |
|  | **DN subset** | **0.001** | **0.002** | **0.006** | **0.062** | **0.020** | **0.048** |
| **CD107a expression** | **Bulk MAIT** | 0.969 | >0.999 | **0.002** | **0.006** | **0.065** | **0.019** |
|  | **CD4 subset** | 0.270 | 0.910 | 0.578 | >0.999 | 0.741 | >0.999 |
|  | **CD8 subset** | 0.280 | 0.753 | **<0.001** | **0.003** | **0.009** | **0.026** |
|  | **DN subset** | 0.565 | >0.999 | **0.003** | **0.013** | **0.007** | **0.017** |
| **IFNγ expression** | **Bulk MAIT** | **0.083** | 0.313 | **<0.001** | **<0.001** | **<0.001** | **<0.001** |
|  | **CD4 subset** | **0.002** | **0.004** | **<0.001** | **0.003** | **0.006** | **0.010** |
|  | **CD8 subset** | **0.046** | 0.117 | **<0.001** | **<0.001** | **<0.001** | **<0.001** |
|  | **DN subset** | **0.064** | **0.038** | **<0.001** | **0.001** | **0.001** | **<0.001** |

**p-value:** p-values for comparisons of MAIT cell frequencies, responses and, activation between respective group and healthy control group. **Adjusted p-value:** p-values for comparisons of MAIT cell frequencies, responses and activation between respective groups and healthy control groups after using Kruskal-Wallis test and Dunn’s test for multiple comparisons.
