# Supplementary material for: Functional and Activation Profiles of Mucosal-Associated Invariant T Cells in Patients With Tuberculosis and HIV in a High Endemic Setting
Source: Front Immunol. 2021 Mar 22;12:648216. doi: 10.3389/fimmu.2021.648216 (PMC8019701; doi:10.3389/fimmu.2021.648216)
Supplement: Supplementary Table 3 — p-values of group comparisons for BCG 5 stimulated MAIT cells, before and after adjusting for multiple comparisons using Dunn's test. [file Table_3.docx]

**Supplementary Table 3: p-values of group comparisons for BCG 5 stimulated MAIT cells, before and after adjusting for multiple comparisons using Dunn’s test.**

|  | **HIV** | | **aTB** | | **HIV-TB** | |
| --- | --- | --- | --- | --- | --- | --- |
|  | **p-value** | **adjusted p-value** | **p-value** | **adjusted p-value** | **p-value** | **adjusted p-value** |
| **CD107a** | 0.548 | >0.999 | **0.005** | **0.015** | 0.250 | 0.964 |
| **IFNγ** | 0.050 | 0.173 | **0.001** | **0.006** | **0.011** | **0.018** |
